# Ultrasound-guided Achilles tendon rupture repair using knotless barbed suture: a cadaveric study

**DOI:** 10.1007/s10396-025-01592-7

**Published:** 2025-10-16

**Authors:** Toru Omodani, Michael Khadavi, Yoshifumi Takatsume, Alexandre Lavigne

**Affiliations:** 1 Tokyo Advanced Orthopaedics, Tokyo, Japan; 2 Kansas City Orthopedic Alliance, Overland Park, KS USA; 3 Department of Physical Medicine and Rehabilitation, University of Missouri, Columbia, MO USA; 4 Department of Anatomy, Keio University School of Medicine, Tokyo, Japan; 5 Faculty of Medicine, Université de Montréal, Montréal, QC Canada; 6 Department of Physical Medicine and Rehabilitation, Hôtel-Dieu du Centre hospitalier de l’Université de Montréal, 3840, Rue Saint-Urbain, Montréal, QC H2W 1T8 Canada

**Keywords:** Achilles tendon rupture, Achilles tendon repair, Barbed suture, Ultrasound, Ultrasound-guided surgery

## Abstract

**Purpose:**

To describe a novel, minimally invasive, ultrasound-guided Achilles tendon repair technique using a knotless barbed suture and to assess its feasibility in a cadaveric model.

**Methods:**

A midportion Achilles tendon rupture was simulated in a Thiel-embalmed cadaver. The tendon was repaired percutaneously under ultrasound guidance using a 0.6-mm USP 1 knotless barbed suture mounted on a 19-gauge, 3.5-inch curved needle. The needle was advanced intratendinously under continuous ultrasound guidance. Tendon reapproximation was assessed with ultrasound and confirmed by anatomical dissection. The resting plantar flexion angle was measured before and after the repair.

**Results:**

The Achilles tendon repair technique resulted in successful reapproximation of the tendon ends with anatomic alignment. Post-repair ultrasound and dissection confirmed accurate intratendinous suture placement and full tendon continuity. The resting plantar flexion angle increased from 23° to 50° after the repair.

**Conclusion:**

This is the first report of an ultrasound-guided Achilles tendon repair using a knotless barbed suture. The technique appears feasible in a cadaveric model and may represent a promising minimally invasive option for patients requiring improved tendon approximation. Its office-based approach may reduce surgical risks associated with conventional repair and tendon elongation seen with conservative treatment. Further biomechanical and clinical studies are warranted to evaluate its safety, durability, and functional outcomes.

**Supplementary Information:**

The online version contains supplementary material available at 10.1007/s10396-025-01592-7.

## Introduction

Midportion Achilles tendon rupture (ATR) is frequently seen in athletes and sedentary individuals, typically occurring during a forceful eccentric contraction of the triceps surae while the ankle is dorsiflexing [1]. It affects men more often than women, with an incidence ranging from six to 37 per 100,000 person-years [2, 3].

Various midportion ATR repair techniques have been described in the literature to create tendon end reapproximation and attachment, including open, mini-open, percutaneous, and minimally invasive device-assisted approaches [4–11]. Although ATR surgical repair reduces rerupture and tendon elongation compared with conservative care, these techniques carry notable complications: infection rates of up to 18% and risks of sural-nerve injury, delayed wound healing, and adhesions [11–13]. Minimally invasive options—such as the Percutaneous Achilles Repair System (PARS) that uses a suture-passing jig—show lower complication rates than open repair, yet the complication risk remains clinically significant [14–16]. Nonetheless, all of these procedures carry risks associated with general anesthesia, require operating room resources, and contribute to higher healthcare costs [17–20].

There is an international trend toward the conservative treatment of midportion ATR with immobilization in plantar flexion, driven by studies showing comparable outcomes with or without surgery [2, 3, 11]. However, athletes often still opt for surgical treatment to minimize the risks of Achilles tendon (AT) rerupture, tendon elongation, and decreased triceps surae strength [21–23]. The tendon elongation and loss of strength seen with conservative treatment may result from incomplete approximation of the tendon ends, which can retract and heal in a lengthened position [24].

An ideal solution for midportion ATR would tightly and precisely approximate the two tendinous ends without the aforementioned complication profile of ATR repair surgery. In this cadaveric study, we describe a novel technique that attempts to fulfill these criteria.

## Methods

### General Design

**Fig. 1:**
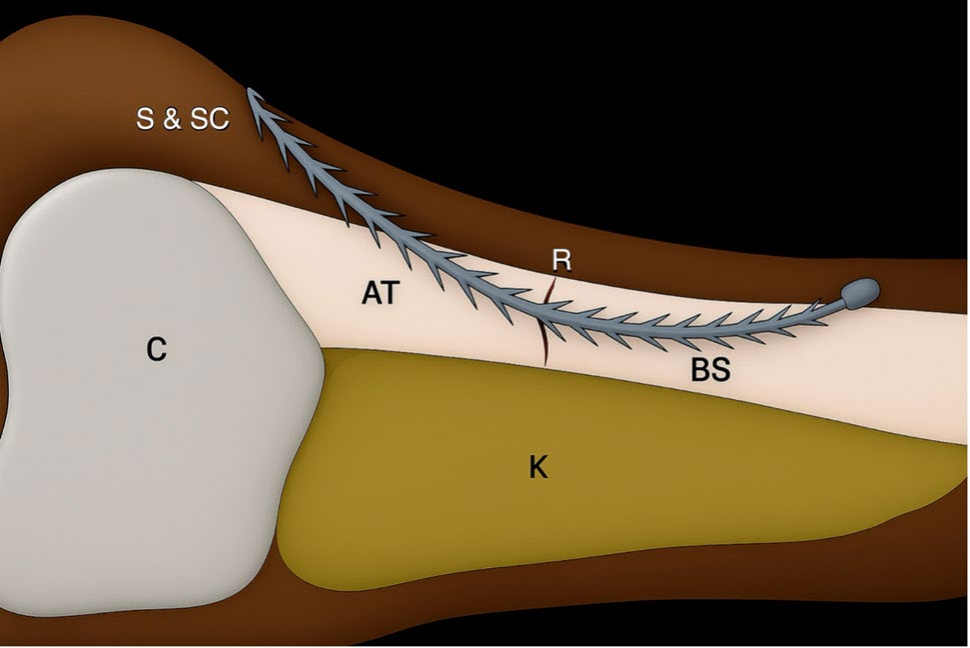
Illustration of the proposed ultrasound-guided Achilles tendon repair technique. AT: Achilles tendon, BS: barbed suture, C: calcaneus, K: Kager’s fat pad, R: midportion Achilles tendon rupture, S and SC: skin and subcutaneous tissue

**Fig. 2:**
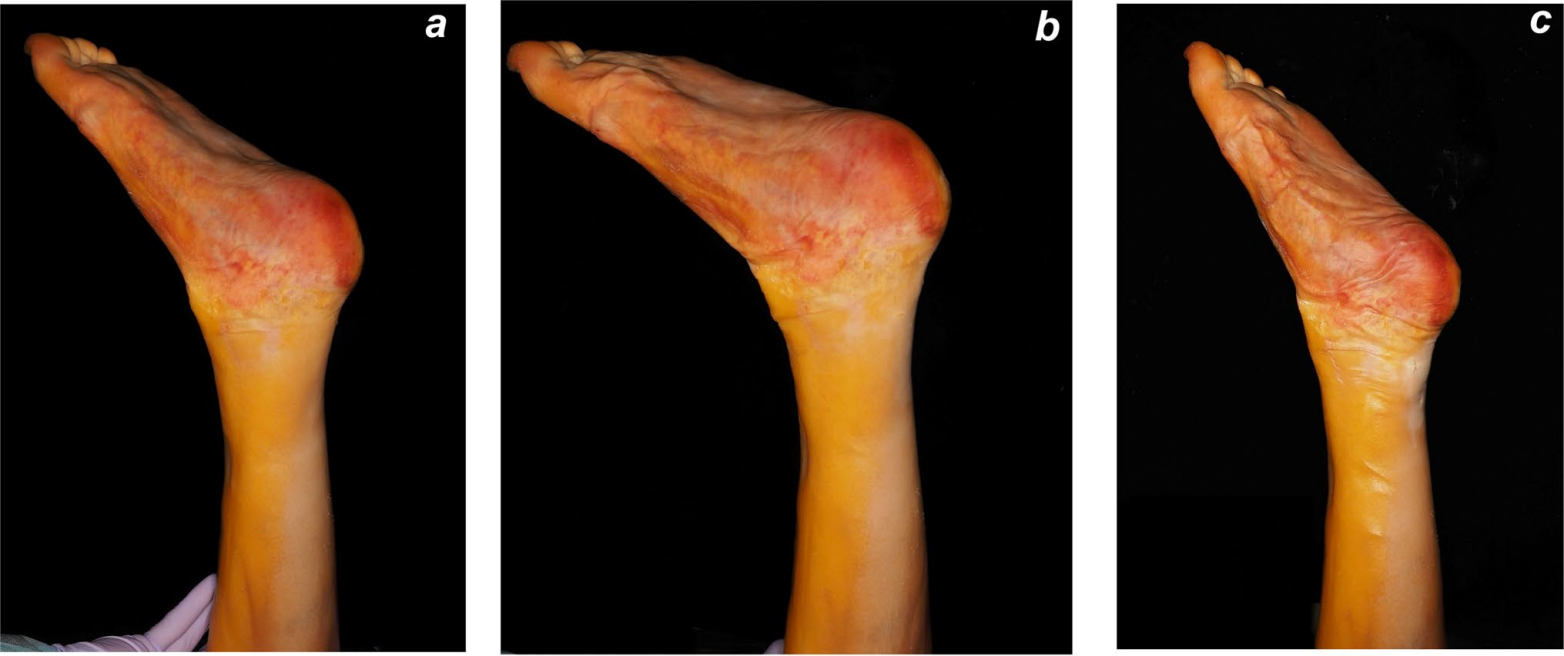
**a**, **b**, **c** Resting plantar flexion angle measurements with the knee flexed to 90° in the prone position: **a** 45° prior to the simulated Achilles tendon rupture; **b** 23° following complete transection of the Achilles tendon; and **c** 50° after completion of the ultrasound-guided Achilles tendon repair

This cadaveric study was conducted to investigate a novel ATR repair using a barbed suture under ultrasound (US) guidance, as imaged in Fig. 1. The study was approved by our Institutional Review Board (approval number: 20070026). A left lower extremity cadaver was used in this study. The individual had, prior to death, declared their intention to donate their body for educational and research purposes. The remains were embalmed using Thiel’s method [25]. All procedures were conducted in accordance with relevant local and international ethical guidelines and laws pertaining to the use of human cadaveric donors in anatomical research [26]. The cadaver specimen was placed in the prone position and secured to the procedural table for all measurements and procedures. The resting plantar flexion angle of the ankle joint with the knee flexed to 90° was measured and recorded as 45° (Fig. 2a).

### Equipment

The procedure was performed using a SONIMAGE HS1 US machine (Konica Minolta, Japan) and a linear 11-MHz transducer. Additional medical equipment included an 11-blade scalpel, a needle holder, one knotless barbed suture (Stratafix Symmetric PDS Plus, USP 1, 0.6-mm diameter; Ethicon, Johnson & Johnson, New Brunswick, NJ, USA), a 19-gauge, 3.5-inch straight needle (TOP Corporation, Tokyo, Japan), and surgical scissors. The needle’s plastic hub was separated from the metal shaft, and the barbed suture was inserted into the tail end of the needle. The junction was crimped with a needle holder to ensure fixation, and the needle was gently curved to facilitate handling (Electronic Supplementary Material).

### Midportion Achilles tendon rupture simulation

**Fig. 3:**
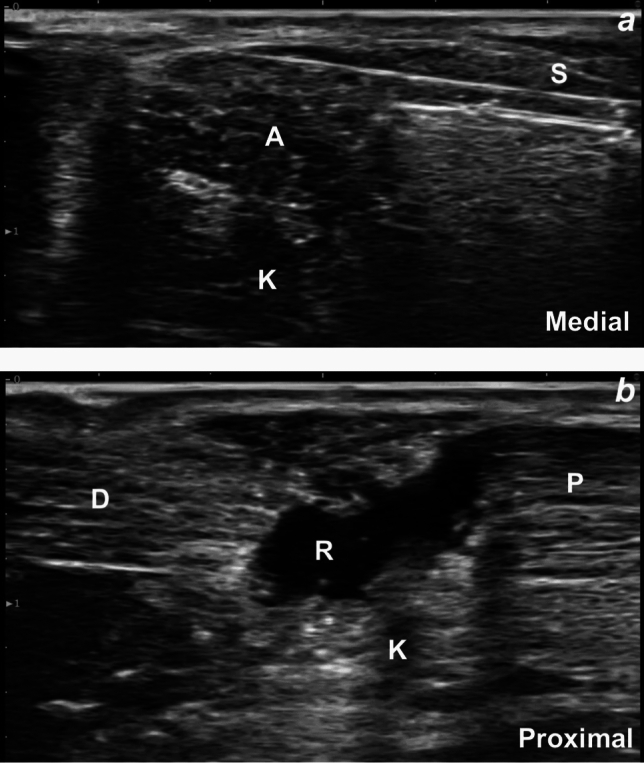
**a**, **b** Ultrasound images illustrating the simulated Achilles tendon rupture: **a** short-axis view showing the scalpel transecting the midportion of the tendon using an in-plane approach; and **b** long-axis view demonstrating gapping between the proximal and distal tendon ends following transection. A: Achilles tendon, D: distal end of the tendon, K: Kager’s fat pad, P: proximal end of the tendon, R: rupture, S: scalpel

The AT was identified in the short-axis view at approximately 4 cm proximal to the insertion on the calcaneus. A scalpel was inserted medially, and the AT was transected in the short-axis direction under US guidance (Fig. 3a). Complete, full-thickness discontinuity was confirmed with a long-axis US view (Fig. 3b). The resting plantar flexion angle decreased to 23° (Fig. 2b), again confirming complete transection.

### Procedural description

**Fig. 4:**
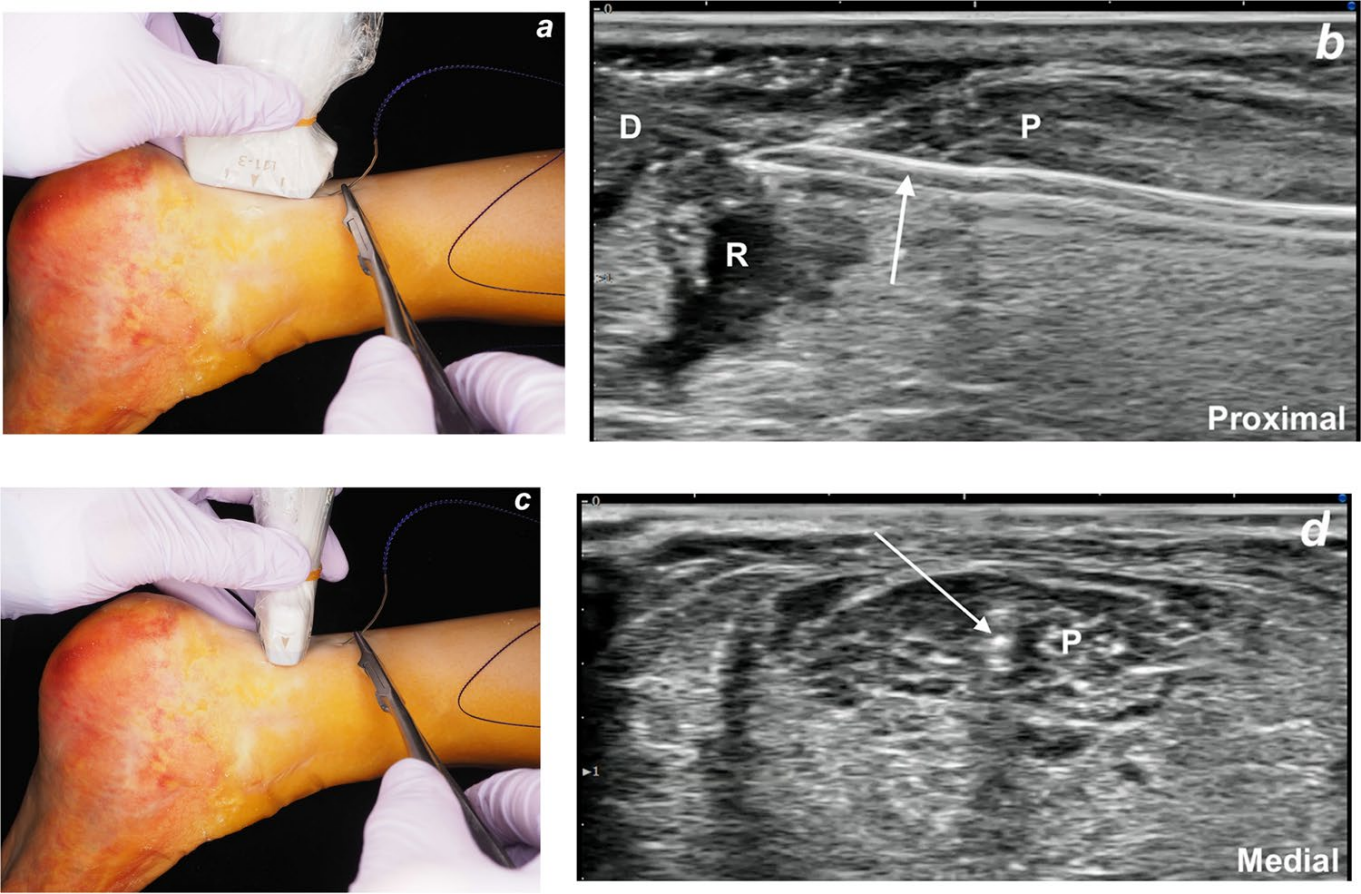
**a**,** b**,** c**,** d** Probe positioning in the longitudinal axis (**a**) with the corresponding ultrasound image showing intratendinous needle advancement (**b**), and probe positioning in the short axis (**c**) with the corresponding short-axis ultrasound image (**d**). Arrow: needle, D: distal end of the tendon, P: proximal end of the tendon, R: rupture site

**Fig. 5:**
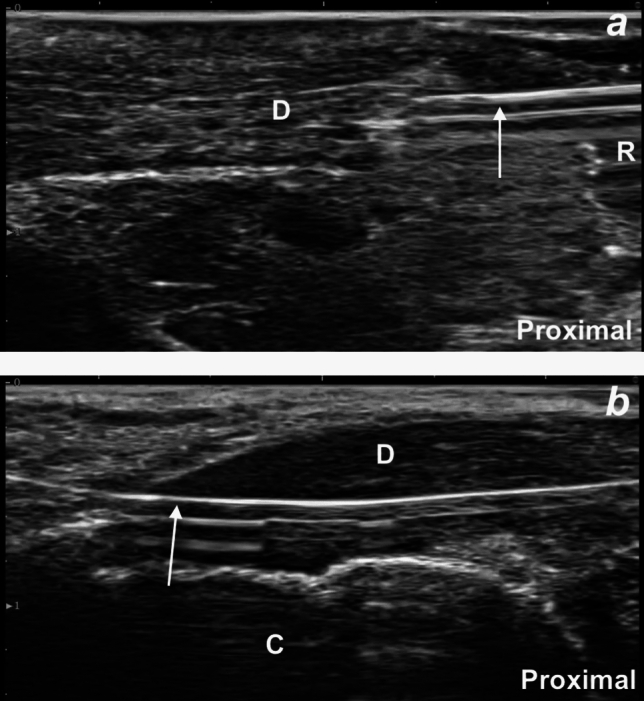
**a, b** Ultrasound images showing the needle advancing through the distal end of the Achilles tendon (**a**) and the needle exiting the tendon (**b**). Arrow: needle, C: calcaneus, D: distal end of the tendon, R: rupture site

**Fig. 6:**
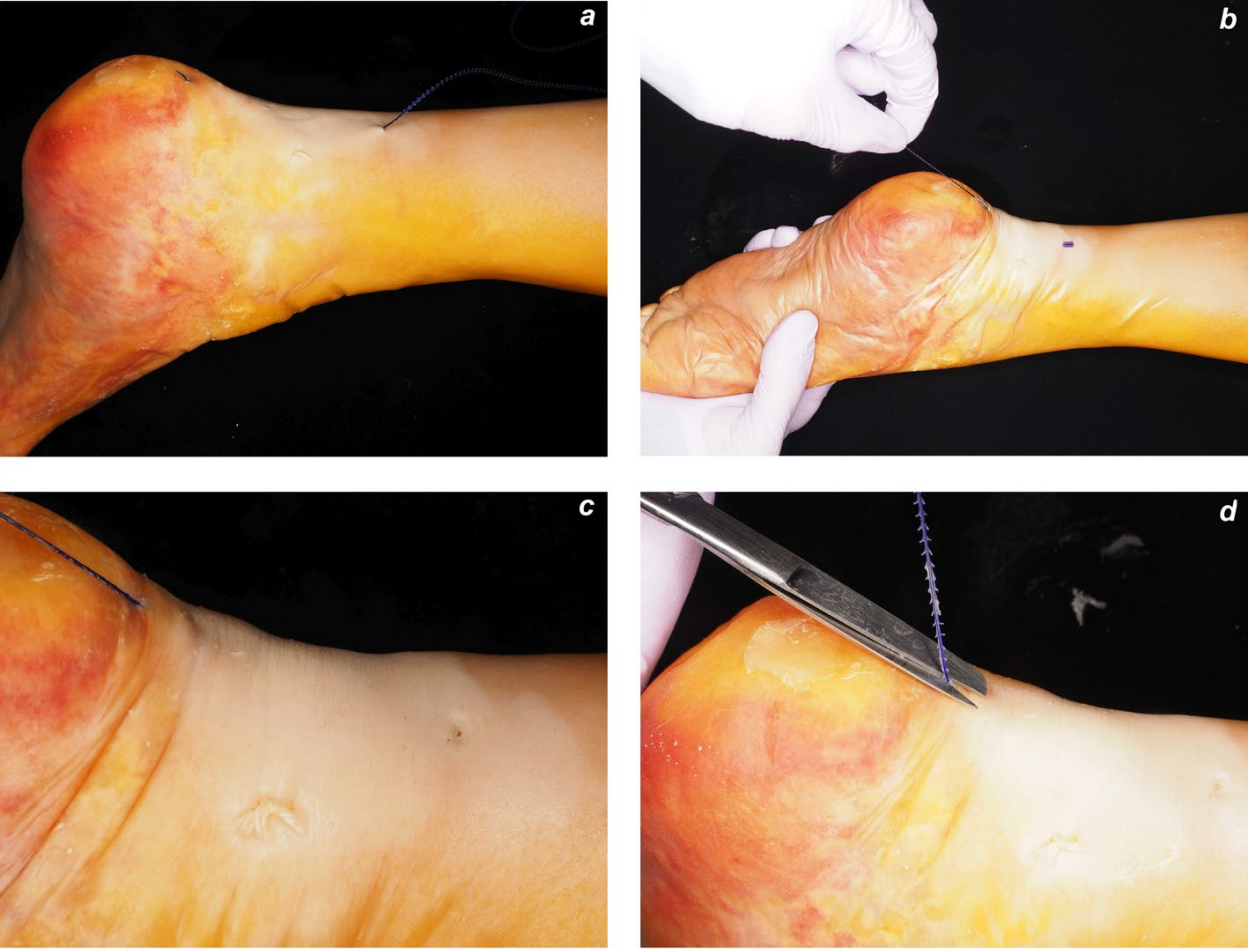
**a**,** d**,** c**,** d** Procedural steps of the ultrasound-guided Achilles tendon repair: **a** the needle emerging from the skin approximately 3 cm distal to the rupture site; **b** the ankle held in maximal plantar flexion to facilitate tendon reduction while applying distal traction on the suture; **c** the barbed suture’s stopper positioned subcutaneously; and **d** cutting of the distal portion of the suture flush with the skin

The ankle was positioned in maximal plantar flexion to approximate the two ends of the AT. US was used to scan the AT and the surrounding structures including the sural nerve. The two ends of the AT were identified. The curved needle was inserted with the needle holder approximately 3 cm proximal to the rupture site, a few millimeters from the proximal end of the ultrasound probe, and midline with respect to the AT. The needle was advanced intratendinously under real-time US guidance, and was continuously visualized in both long and short axes to ensure a central intratendinous path (Fig. 4). It was then passed through the distal tendon end (Fig. 5). No instrument was used to stabilize the distal tendon end during needle advancement as it remained in position, likely due to containment by the paratenon and its distal attachment to the calcaneus. The needle was exteriorized approximately 3 cm distal to the rupture site. The barbed suture was then pulled out of the skin (Fig. 6a). The suture was tightened by applying plantar flexion to the ankle while pulling on the distal end (Fig. 6b). A 3-mm skin incision was made with the scalpel to embed the barbed suture’s stopper on the Achilles paratenon surface (Fig. 6c). The suture was then secured by tensioning it appropriately. Once appropriate tension was achieved, the distal portion of the suture exiting the skin was cut flush with the skin using surgical scissors (Fig. 6d).

## Results

**Fig. 7:**
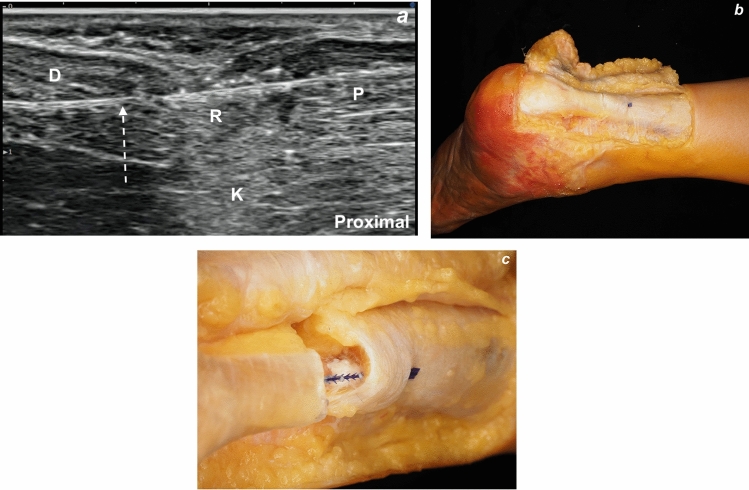
**a**, **b**, **c** Confirmation of tendon ends approximation following ultrasound-guided repair. **a** Ultrasound image of the Achilles tendon in the longitudinal axis, obtained prior to dissection, showing appropriate approximation of the tendon ends without residual gapping. **b** Cadaveric image of the dissected Achilles tendon in a resting position, showing that the tendon ends were in direct contact, confirming successful reapproximation. **c** Intratendinous dissection showing that the suture was accurately positioned in the middle of the tendon substance. P: proximal end of the tendon, D: distal end of the tendon, R: rupture site, K: Kager’s fat pad, dashed arrow: barbed suture

US examination confirmed that the tendon ends were well approximated and restored to continuity (Fig. 7a). After fixation, the resting plantar flexion angle was reassessed and found to be 50° (Fig. 2c), indicating restoration of objective tension on the tendon.

An open dissection was performed to directly evaluate the repair site. After skin and subcutaneous tissue reflection, the AT surrounded by the paratenon was exposed. The tendon ends were in direct contact, and the repair remained intact even with mild dorsiflexion stress (Fig. 7b). Moreover, the suture was accurately positioned within the tendon intrasubstance, confirming that the US-guided technique allowed precise intratendinous passage (Fig. 7c).

## Discussion

This is the first description of a percutaneous, US-guided AT repair using a knotless barbed suture. Our approach may offer advantages over both traditional surgical and conservative treatments, with potential to improve outcomes for patients with ATR.

This US-guided repair may reduce surgical complications, particularly that of infection, as the skin incision is just 3 mm [27]. In addition, the procedure can be performed in an office-based setting with a sterile procedural room, eliminating the need for an operating room and its associated costs. The minimally invasive nature of the technique allows for regional anesthesia using tibial and common peroneal nerve blocks, a so-called “popliteal block,” with US guidance [28]. These nerve blocks can be performed by the same physician performing the tendon repair. This approach also avoids transverse suture passage through the tendon, minimizing the risk of sural nerve injury that is not uncommon in open techniques [4]. Continuous visualization of neurovascular structures in US-guided soft-tissue procedures yields an excellent safety profile; moderately severe complications occur in 0.1% of other US-guided soft tissue procedures, with no reported severe, life-threatening complications [29].

A variety of ATR repair techniques have been described, including open, mini-open, percutaneous (e.g., Ma & Griffith), device-assisted (e.g., PARS), and more recently, US-guided approaches [5–7, 9, 10, 30–35]. In comparison, our technique offers several advantages: a smaller incision, no suture passage through the transverse plane of the AT, and no operating room or anesthesiologist involvement, as shown in Table 1. These aspects of the knotless, barbed-suture technique may further minimize complications.

**Table 1:** Comparison of Achilles tendon rupture repair techniques

Technique	Need for operating room and general anesthesia	Incision size	Suture passage type	Complication rate	References
Open repair	Yes	8–12 cm	Transverse, multiple passes	15.5%, more specifically, more wound complications including infection	[6–9, 11, 32]
Percutaneous repair (e.g., Ma & Griffith)	Yes	1–2 cm	Transverse, 6–8 passes	10–12%, fewer wound complications, but higher sural nerve injection risk	[6–9, 11, 32]
Mini-open repair	Yes	2–4 cm	Transverse, multiple passes	10.4%, mostly minor complications, very low risk of infection and sural nerve injury (< 1%)	[10, 35]
Device-assisted repair (e.g., PARS)	Yes	1–2 cm + device ports	Transverse, 6–8 device-guided passes	10.4%, mostly minor complications, very low risk of infection and sural nerve injury (< 1%)	[11, 15]
US-guided percutaneous repair	Yes	1–2 cm	Transverse, multiple US-guided passes	8–10%, US guidance might help reduce sural nerve injury risk	[5, 30, 34]
US-guided barbed suture (present technique)	No	3 mm	Longitudinal plane, 1 US-guided pass	Unknown, likely extremely low	Not applicable

Given the near absence of theoretical contraindications, this percutaneous, US-guided technique could serve as a primary surgical option for midportion ATRs. Clinical data will be needed to confirm the long-term biomechanical properties of the tendon with this technique, such as plantarflexion strength. Moreover, the optimal timing for return to sport following this technique remains unknown, and comparative studies with open repairs using non-absorbable, high-strength constructs will be valuable.

An absorbable suture was selected for this technique due to its potential to reduce complications such as tendon pain, soft tissue irritation, and intratendinous granuloma formation—issues more commonly associated with retained non-absorbable suture material in ATR repairs [6, 36, 37]. Two randomized controlled trials comparing absorbable and non-absorbable sutures—using both open and device-assisted repair techniques—found no significant differences in plantar flexion strength, functional outcomes, or rerupture rates [37, 38]. Although non-absorbable sutures possess greater theoretical tensile strength according to biomechanical in vitro studies [39], clinical studies have not demonstrated superior performance [40], possibly because the tensile strength difference is not clinically significant during the early phases of tendon healing.

The absorbable, knotless suture used in this technique retains its maximal tensile strength for up to 6 weeks, which may provide adequate mechanical stability during the early healing phase [41]. This temporary fixation could require a shorter immobilization period than the standard 8-week protocol used in conservative management since the tendon ends are directly reapproximated [42]. Moreover, this treatment could potentially reduce tendon elongation and preserve plantar flexion strength—both of which are common concerns with conservative management of ATR.

The primary limitations of this study were that the procedure was performed on a single cadaver and that no biomechanical measurements were assessed, such as load-to-failure testing. The next logical step would be to conduct a prospective clinical study to evaluate feasibility, safety, and functional outcomes in patients who opt for this new US-guided procedure with an appropriate recovery protocol.

## Conclusion

This US-guided ATR repair using a barbed suture in a cadaveric model demonstrates the feasibility of this technique for midportion ATR. The proximal and distal tendon ends were successfully reapproximated with anatomic alignment. This minimally invasive repair may minimize surgical risks while reducing the likelihood of tendon elongation and plantarflexion weakness—common complications of conservative treatment for ATR.

## Supplementary Information

Below is the link to the electronic supplementary material.

Electronic Supplementary Material: Knotless barbed suture attached to a gently curved 19-gauge, 3.5-inch needle (JPG 194 KB)

## Data Availability

All data generated or analyzed during this study are included in this published article. No additional data are available.
