# Supplementary figures and images for: Ultrasound-guided Achilles tendon rupture repair using knotless barbed suture: a cadaveric study
Source: J Med Ultrason (2001). 2025 Oct 16;53(2):227–34. doi: 10.1007/s10396-025-01592-7 (PMC13092523; doi:10.1007/s10396-025-01592-7)

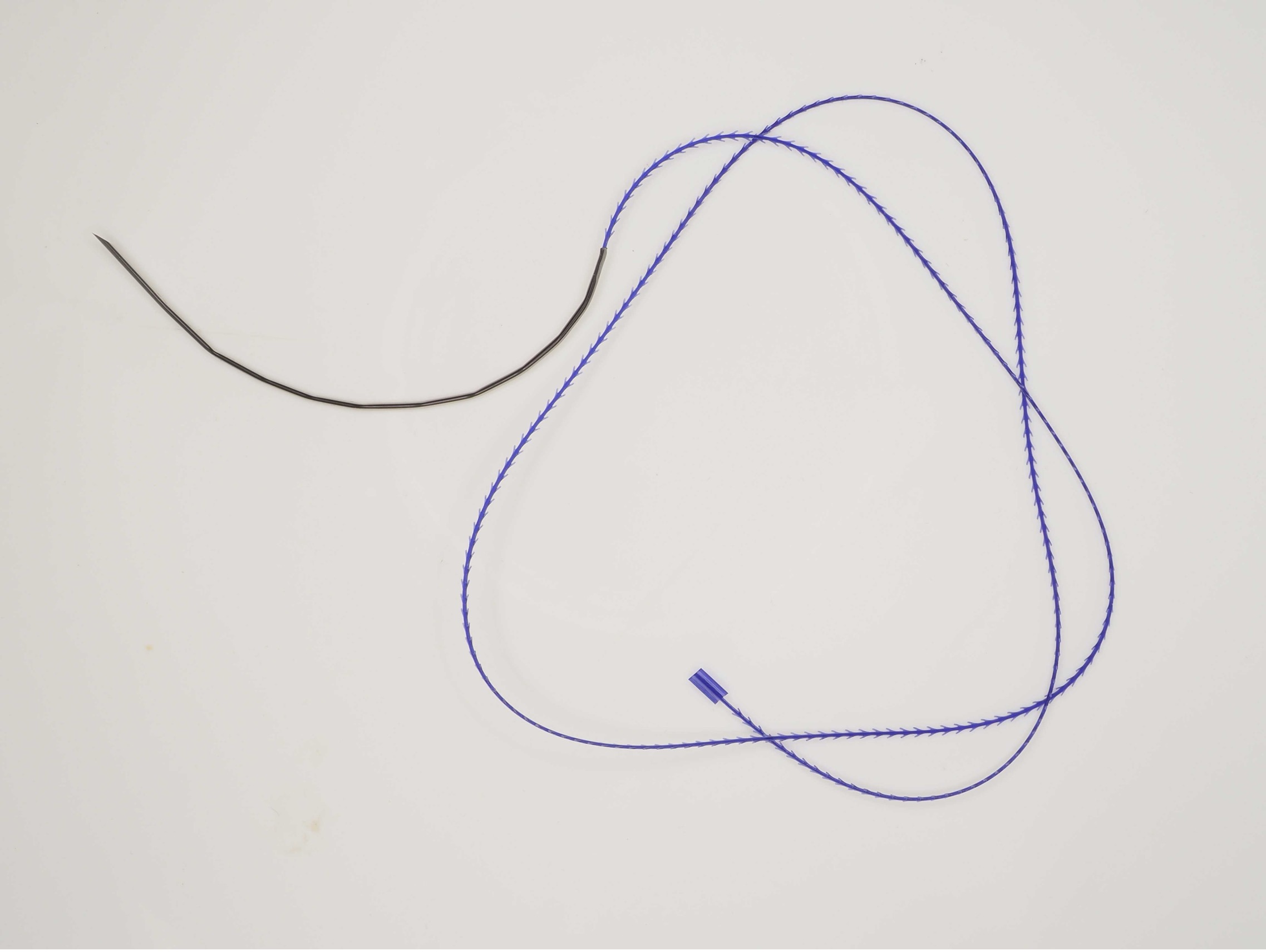

Supplement: Supplementary file 1 — Electronic Supplementary Material: Knotless barbed suture attached to a gently curved 19-gauge, 3.5-inch needle (JPG 194 KB) [file 10396_2025_1592_MOESM1_ESM.jpg]
